# COVID-19 Lockdown and Self-Perceived Changes of Food Choice, Waste, Impulse Buying and Their Determinants in Italy: QuarantEat, a Cross-Sectional Study

**DOI:** 10.3390/foods10020306

**Published:** 2021-02-02

**Authors:** Alessandro Scacchi, Dario Catozzi, Edoardo Boietti, Fabrizio Bert, Roberta Siliquini

**Affiliations:** 1 Department of Public Health Sciences and Paediatrics, University of Torino, 10126 Torino, Italy; alessandro.scacchi@unito.it (A.S.); dario.catozzi@unito.it (D.C.); edoardo.boietti@unito.it (E.B.); roberta.siliquini@unito.it (R.S.); 2 City of Health and Science of Turin, Azienda Ospedaliero-Universitaria, 10126 Torino, Italy

**Keywords:** lockdown, COVID-19, coronavirus, food choice, food purchase, food waste, impulse buying, food consumption, mental health, emotional eating

## Abstract

Data about self-perceived food choice (FC) changes and their determinants during COVID-19 lockdowns are limited. This study investigated how the Italian lockdown affected self-perceived food purchases (FP), occurrence of impulse buying (IB), household food waste production (HFWP) and their determinants. A web-based cross-sectional survey was distributed in May 2020, collecting an opportunistic sample of the Italian population. A total of 1865 (70% females) people were enrolled, the median age was 29 (IQR 16.0). Most of the sample increased overall FP (53.4%), food consumption (43.4%), reduced HFWP (53.7%) and halved the prevalence of IB (20.9%) compared to the period before the lockdown (42.5%). Baking ingredients, fresh vegetables, fresh fruit and chocolate had the largest sales increase by individuals, while bakery products, fresh fish and salted snacks purchases highly decreased. Increased FP was associated with the occurrence of IB (adjOR 2.48, *p* < 0.001) and inversely associated with not having worked during lockdown (adjOR 0.71, *p* = 0.003). Multivariable logistic regressions revealed occurrence of IB was associated with low perceived dietary quality (adjOR 2.22, *p* < 0.001), resulting at risk, according to the Emotional Overeating Questionnaire (EOQ, adjOR 1.68, *p* < 0.001), and inversely associated with decreased HFWP (adjOR 0.73, *p* < 0.012). Reduced HFWP was associated with higher perceived dietary quality (adjOR 2.27, *p* < 0.001) and negatively associated with low score at WHO-5 Well-Being Index (adjOR 0.72, *p* = 0.002). The Italian lockdown highly affected FC behaviours, leading to positive and sustainable habits towards food purchase and consumption. Public health interventions are needed to keep these new positive effects and avoid negative consequences in case of future lockdowns.

## 1. Introduction

On 21 February 2020, the first case of indigenous SARS-CoV2 infection in Italy was reported. A few days later, the lockdown was established in some provinces of northern Italy [1,2]. On 9 March 2020, the Italian Government decided for a stringent containment measure of lockdown on the entire national territory [3]. This measure was effective in flattening the epidemic curve and bought valuable time, allowing for the number of intensive care beds to be nearly doubled before the National Health System reached maximum capacity [4]. During lockdown, people could leave their homes only for primary activities such as work in key sectors of industry, care and services, physical exercise, medical care or food shopping. On 3 May, the government declared the end of the first phase of the lockdown by introducing a series of less restrictive anti-contagion rules [5].

The global pandemic of COVID-19 has caused radical changes in the structure of people’s daily routines in most of the countries around the world, including the way people buy food, that has changed dramatically [6]. In the weeks immediately preceding the Italian lockdown, people began to panic-buy and stockpile essential and non-perishable products such as water, gloves, carbohydrate-rich staples (e.g., bread, pasta), canned food, hand sanitisers, and even toilet paper [6]. On a national scale, in March, during the acute phase of the lockdown, a +17% of grocery sales was reported, reaching almost EUR 6 billion, EUR 860 million more than the same period during the previous year. Purchase choices were mostly directed toward the stocking of non-perishable foods, in order to face potential scarcity situations. The increase in purchases affected moreover pasta, UHT (ultra-high temperature) milk, canned fish, flours and eggs, frozen foods, cold cuts and parmesan, and water [7]. Neighbourhood shops were preferred over hypermarkets, due to large queues and proximity [8]. A similar trend affected online shopping, reaching virtual overcrowding and service outages [9]. The major increase in purchases occurred in South Italy, despite being the least affected territory by COVID-19 [8].

These data are not surprising. In the literature, indeed, it is well known that during home confinement people tend to increase their food intake [10,11]. A quarter of the Italian population consumed more food and one third increased time spent cooking at home [12], while an Italian study showed that half of the sample felt anxious about their eating habits, consumed comfort food and were inclined to increase food intake to feel better [13]. Furthermore, during lockdown the perception of weight gain was observed in almost half of an Italian sample and young people resulted having a higher adherence to the Mediterranean diet [14]. Another study, conducted in Poland, reported that during quarantine people ate more snacks [15]. In particular, those with a high BMI (body mass index) tended to introduce less vegetables, fruit and beans in their diet, while a greater amount of alcohol and tobacco consumption was reported [15]. The reported big changes in food purchase and consumption habits, such as the increased reuse of leftovers, could have affected the production of household food waste, as reported in a Tunisian study [16].

However, evidence about changes of food choice, household food waste production and their associated factors during lockdown in Italy is poor. Existing studies have been carried out on limited samples or have collected data for short periods, in the primeval phase of the lockdown. Hence, it is important to increase our knowledge on the self-reported change of habits that occurred during home confinement, to encourage proactive strategies in view of potential future lockdown measures and to keep any new positive behaviours toward maintaining a sustainable and healthy lifestyle in the future.

The aim of this study was to investigate, during lockdown, how Italian people have perceived the change of their food purchases and eating habits and what are the factors associated with the self-perceived increase in food purchases, occurrence of impulse buying and household food waste production. To date, this is the first study investigating the impact of the lockdown on these habits in a national sample.

## 2. Materials and Methods

### 2.1. Study Design and Questionnaire

The QuarantEat study investigated how a sample of Italian inhabitants was affected by the lockdown in terms of self-perceived variations of food purchase, food consumption habits, physical activity levels and how home confinement impacted on mental well-being as well as on the presence of emotional overeating.

An online survey was developed using the Uniquest (LimeSurvey) platform, which was made available by the University of Turin. Our questionnaire was spread among the Italian population through a web link shared by institutional social media pages and the personal accounts of researchers. This procedure led to the enrolment of an opportunistic sample of citizens. The survey was spread a few days after the end of the Italian lockdown, starting from May 6th, in order to highlight the effects of the whole home confinement experience on people’s habits and behaviours. The enrolment ended on the 31 of May, some weeks after the end of the lockdown.

The research protocol was approved by the Ethical Commission of University of Turin (prot. no. 197989). Inclusion criteria were: age equal or older than 18 years, living in Italy during lockdown period, being able to give informed consent to enrolment in the study in Italian. Before starting the questionnaire, each participant was shown a brief written summary including the aims of the research project, and finally each of them confirmed the enrolment to the study declaring their informed consent.

The questionnaire consisted of 40 questions, divided into 6 sections: socio-demographic assessment, physical activity, food purchase habits, food consumption behaviours, mental well-being evaluation and occurrence of emotional overeating. Two validated tests were included: the 5-item World Health Organization Well-Being Index (WHO-5) questionnaire and the Emotional Overeating Questionnaire-5 (EOQ-5). The full version of the questionnaire, translated into English, is available as a Supplementary Materials.

The socio-demographic section included personal data (age, gender, smoking status, relationship status, offspring) and a variety of items regarding the living environment, such as housing place, the presence of a backyard, cohabitation, geographical context (region of Italy) and the working condition during home confinement. Self-reported height and weight were included to calculate BMI. Regions of Italy were later gathered in three geographical areas as advised by National Institute of Statistics (ISTAT): North, Centre, South and Isles. Physical activity (PH) habit was investigated by asking if exercises were practiced during and before lockdown.

Overall self-perceived food consumption changes during lockdown were assessed, as well as the quality of diet and food waste, in terms of subjective increased, decreased or unvaried during lockdown compared to the period before. In addition, diet was investigated, intended as every eating regime with the purpose of body control (weight loss or gain, high protein diets), or medical reasons (due to allergies and food intolerance). Finally, we asked if, during lockdown, on an everyday basis, time spent cooking increased, decreased, or remained unchanged.

The place of food purchase was investigated (supermarket, discount, market, neighbourhood shop, online shop, home delivery), along with shopping frequency in terms of overall times leaving home for buying food per week (the Italian government suggested to go shopping no more than once per week) [17]. Impulse buying behaviour was assessed by asking if any sense of guilt or unnecessary purchase occurred after grocery shopping during lockdown, and if it ever happened before the lockdown. Finally, we proposed a list of 50 foods asking whether their purchase increased, decreased or unchanged during lockdown, as well as if it has never been bought.

To evaluate the impact of home confinement on mental health and psychological well-being in people living in Italy immediately after the lockdown, a section of the survey included the WHO-5 questionnaire, validated in Italian language and used worldwide in research [18]. It can be used as a sensitive and specific screening tool for risk of depression. This questionnaire contains five non-invasive statements about feelings during the last 14 days. A WHO-5 cut-off score of ≤50 is recommendable for screening for clinical depression [18].

To evaluate the occurrence of Emotional Overeating during lockdown as a coping mechanism, a section of the survey included the EOQ-5 questionnaire, validated in Italian language [19]. The EOQ-5 is a brief, valid and reliable 5-item self-report that measures the frequency of overeating behaviour in response to five negative emotions (anxiety, sadness, loneliness, tiredness and anger) during the last 28 days. A cut-off score of 2 points identifies individuals at risk for binge eating disorders. Higher EOQ-5 scores are associated with higher risk of binge eating, lower mental well-being, and lower mindful eating [19].

### 2.2. Statistical Analysis

Overall descriptive analyses were performed for the most prominent variables, showing frequencies for categorical variables and medians and interquartile range (IQR) for scalar variables since the normality Shapiro–Wilk test proved a non-normal distribution for age and shopping frequency. Data were also shown divided in a geographical fashion; Chi-squared test or nonparametric Mann–Whitney or Kruskal–Wallis Tests were performed.

Logistic regression analysis was performed to evaluate determinants of three prominent phenomena, highlighted by collected data and supported by evidence: increased food purchase, occurrence of impulse buying and reduction in household food waste production.

While the vast majority of variables were included in the models unchanged, for analytical purposes some of them were aggregated: for example, education level was dichotomised, aggregating university degree and post-doc studies into high level and the remaining values as middle-low level. WHO-5 and EOQ-5 scores were also dichotomised based on validated threshold values.

The selection of independent variables included into the regression models was achieved with a stepwise backwards method, in which three covariates were protected from exclusion: age, gender and education, since their potential exclusion in the final models could have led to highly biased outcomes. Results were expressed as adjusted odds ratios (AdjOR) and their 95% confidence intervals (95% CI). The statistical significance threshold was set at *p* < 0.05. The software employed for the analysis was IBM SPSS Statistics (Version 25.0). Cases with missing values were excluded from logistic regressions (listwise deletion) and retained in the descriptive analysis (pairwise deletion).

## 3. Results

### 3.1. Participant Characteristics

A total of 2524 individuals began the online survey, and 1923 of them completed every item displayed. Fifty-eight records were excluded due to inclusion/exclusion criteria: 26 of them revealed to be aged 17 years old or younger, while 32 people spent the lockdown period outside the country, reducing the number of eligible records to 1865. An analysis of completion time revealed median duration was 9:25 min (IQR 4:05); since no record could be highlighted as an outlier, none of them were discarded.

Among the sample, 69.9% of participants were female, and the median age was 29 (IQR 16.0), and almost half of them lived in northern Italy (49.7%). People in our sample living in the northern regions most commonly resulted in being women (*p*-value = 0.021) and older (*p*-value < 0.001). Almost an equal number of responders stated having reached the educational level of high school (43.1%) and university degree (42.1%), but with an important geographical variability (*p*-value = 0.006).

The majority of our sample resulted in living with a partner or family (81.8%), with 11.7% living alone and 6.5% with one or more roommate(s). In addition, this variable resulted in an uneven geographical distribution, with fewer people living alone or with cohabitants in the south (*p*-value = 0.002)

Regarding housing, 60.8% of respondents live in a flat or apartment, and 32.9% in an independent house. Living in an independent house was more common in the south (*p*-value = 0.005). One third of our sample, regardless of housing, stated to have in use a private yard or garden.

Only 56.7% of our sample actually worked during lockdown, with a maximum of 62.6% in the north and a minimum of 44.4 in south (*p*-value<0.001). There was a similar geographical distribution for healthcare workers, with a 20.3% in the northern regions, 17.7% in the centre and 14.2% in southern ones (*p*-value = 0.040), and an overall representation of 18.3%. Approximately one-fifth (21.9%) of the sample stated to regularly smoke, slightly more in the south (22.3%, *p*-value = 0.033).

The WHO-5 survey revealed a significant number of respondents (42.4%) potentially at risk of depression development, and 50.7% displayed the occurrence of a significant number of episodes of emotional overeating. None of these scores show asymmetric geographical distribution.

In addition, more than half of our sample claimed to have practiced physical activity during lockdown and as many as 76.2% of participants followed some kind of dietary regimen. Additional descriptive data are provided in Table 1.

### 3.2. Food Purchase Habits

Regarding food purchase habits (Table 2), the majority of our sample increased food purchases (53.4%), while 7.2% reduced them. Food consumption increased in 43.4% of the respondents. A similar size of the sample (46.5%) improved the perceived nutrition quality, while one quarter (26.6%) worsened it. More than half respondents (53.4%) reduced household food waste production. A similar amount of people (55.1%) increased time spent cooking at home, but with smaller increments in the south (*p*-value < 0.001).

People went shopping once or less per week in 68.9% of cases, and the mean was 1.51 (SD 1.31) occasions. A significant geographical difference was found for this behaviour, reaching its peak in southern Italy and Islands (*p*-value = 0.003). Most grocery shopping was made directly by the respondents (81%), showing significant geographical differences: 83.3% in the north, 81.5% in the centre and 74.8% in the south (*p*-value = 0.003).

Impulse buying before the lockdown occurred in 42.5% of the sample, while a strong reduction happened during lockdown; just the 20.9% of respondents occurred in this behaviour, halving its prevalence by 51%.

Delivered food was chosen by 16.6% of the sample, showing a strong North–South geographical gradient, from 18.8% to 11.9% (*p*-value = 0.011).

Finally, a majority of purchases were made in supermarkets (86.7%), while 14.9% of the respondents used online shopping, with great geographical diversities (*p*-value < 0.001), as well as for market purchases, occurring in 8.7% of the sample and being largely more common in the north (*p*-value < 0.001).

### 3.3. Food Purchase Trends

**Figure 1:**
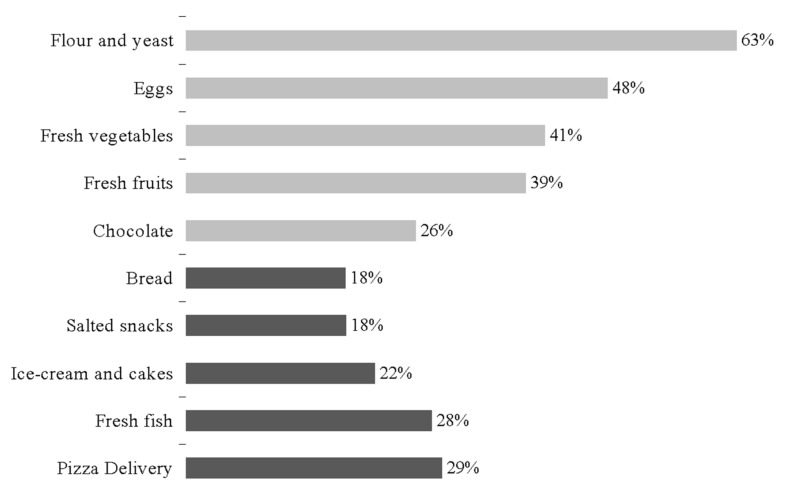
Top five increased (light grey) and top five decreased (dark grey) foods purchased.

Regarding food purchase trends, a selection of the most increased and decreased foods is shown in Figure 1. Baking products and fresh healthy food had the largest sales increase by individuals (flour and yeast +63.2%, eggs +48.4% fresh vegetables +41.2%, fresh fruits +39.0%) as well as chocolate (+26.4%) as indulgence food. Large increases affected pasta and UHT milk, too. The largest individual purchase decreases affected bakery products (pizza delivery −29.4%, ice-cream and cakes −21.7%, bread −18.3%), highly perishable foods (fresh fish −28.2%) and salted snacks (−18.4%). The complete list of purchases is available as a Supplementary Materials.

### 3.4. Determinants of Changes of Food Purchase, Household Food Waste Production and Occurrence of Impulse Buying

Multivariable analysis final models are shown on Table 3. Due to only a small number of participants identifying themselves as “Non-binary” gender (n = 3), this category was unable to be analysed and eventually removed for logistic regression model.

The first model was designed to find associated factors of increased food purchase among population, and the strongest one resulted to be the occurrence of impulse buying (adjOR 2.48, *p*-value < 0.001) followed by increased time spent cooking (adjOR 2.12, *p*-value < 0.001), presence of offspring (adjOR 1.76, *p*-value = 0.0101), perceived nutrition quality (less healthy adjOR 1.66, *p*-value < 0.001; healthier adjOR 1.29, *p*-value = 0.033), while protective factors were being single (adjOR 0.78, *p*-value = 0.028), not having worked during lockdown (adjOR 0.71, *p*-value = 0.003) and younger age (adjOR 0.98, *p*-value = 0.002).

The occurrence of impulse buying during lockdown was positively associated with increased food purchase (adjOR 2.72, *p*-value < 0.001), low perceived quality of diet (adjOR 2.22, *p*-value < 0.001), living alone (adjOR 1.89, *p*-value = 0.002), resulting overweight (adjOR 1.44, *p*-value = 0.024), time spent cooking (decreased adjOR 1.58, *p*-value = 0.039; increased adjOR 1.36, *p*-value = 0.034) high score in EOQ-5 survey (adjOR 1.68, *p*-value < 0.001) and a low score in WHO-5 questionnaire (adjOR 1.73, *p*-value < 0.001), while the only protective covariate resulted to be a decrease in household food waste production (adjOR 0.73, *p*-value = 0.012).

The last model estimates the determinants of decreased household food waste production, finding in healthier perceived nutrition quality the strongest positive predictor (adjOR 2.27, *p*-value < 0.001) and in male gender the strongest negative predictor (0.59, *p*-value = 0.002). Additionally age (adjOR 1.01 per year, *p*-value = 0.006), not working during lockdown (adjOR 1.30, *p*-value = 0.024), smoking (adjOR 1.80, *p*-value < 0.001), BMI different than normal (underweight adjOR 0.71, *p*-value = 0.009; overweight adjOR 1.37, *p*-value = 0.017, obese adjOR1.63, *p*-value = 0.034), time spent cooking (adjOR 1.52, *p*-value < 0.001), WHO-5 score below 50 points (adjOR 0.72, *p*-value = 0.002), impulse buying (adjOR 0.73, *p*-value = 0.013), decreased food purchased (adjOR 1.69, *p*-value = 0.011) had statistically significant results.

## 4. Discussion

Home confinement during lockdown caused strong self-perceived changes in the food purchasing habits and behaviours of Italian residents. The majority of our sample perceived to have increased overall food purchase, food consumption and improved diet quality, reducing household food waste production, increasing time spent cooking at home and halving the prevalence of impulse buying.

Most of our sample followed Italian government suggestions about shopping frequency [17], limiting it to once or less per week, as found in other studies [16,20]. The lowering of shopping frequency was possible by concentrating most purchases in one time and at one place. Indeed, most food purchases occurred in supermarkets, as shown in other studies (64.3% and 75.8%, main frequencies) [16,20]. A big group of purchases were made in neighbourhood shops, where a +40% of sales was registered in April 2020, compared to last year, as well as a +23% compared to March 2020 [21], whilst a remarkable percentage of purchases were made online. The severe restrictions to movements and the presence of long queues out of supermarkets could have discouraged many customers, causing a shift of choice from hyper/supermarkets toward online or small neighbourhood shops. For these reasons, 27.6% of the customers changed their trusted store during lockdown [22]. Expectedly, a minimum number of purchases was performed in street markets, due to strong limitation of them or closure during lockdown.

The large increase in baking products purchases (flour/yeast, eggs, butter and fresh cheese) reflected the increase in self-production and consumption of foods such as pizza, homemade desserts and bread [14] that many people experimented with during lockdown [20,21,23]. Moreover, there was an increase in Google searches for recipes and baking [20]. Conversely, among the most decreased food purchases in our sample were delivered pizza, bread, ice cream and cakes. These foods could have been prepared at home instead of being bought. Actually, most of the sample increased time spent cooking, as an attempt to face boredom for the interruption of the work routine [14], less availability of out-of-home food, up to entertainment of children at home [24], resulting in a positive effect of home confinement. Indeed, home cooking is a healthy habit, related to better dietary quality, lower adiposity and greater adherence to Dietary Approaches to Stop Hypertension (DASH) and Mediterranean diets [25].

The recourse to foods for coping with stress and anxiety could have caused the increase in purchases of chocolate and biscuits [20,23,26]. Indeed, chocolate is also consumed as a stress relief, causing improvement of mood, but at the same time is related with emotional eating [27]. Furthermore, during lockdown there was an augmented prevalence of sleeping disturbances, depressive and anxiety symptoms in Italy [28]. Our results confirmed this trend, indeed the lockdown impacted on the mental health of a critically high portion of our sample. Almost half had a score of ≤50 in the WHO-5 questionnaire, resulting in low mental well-being and being at high risk of depression development [18]. Moreover, there was a high occurrence of emotional overeating in most of our sample during home confinement period, leading to a pathologic relation with nutrition, as an enormous palliative response to negative feelings. Our findings raise the need for public health interventions to take care of these people and to block the development of heavier mental health issues that can last after the pandemic as psychological aftermaths.

The rise of purchases of shelf-stable foods is typically associated with emergencies and uncertain times, even suggested by the government in the USA [29]. High increases of UHT long shelf-life milk and pasta purchases were reported in our samples and, in several articles [8,21,26], giving witness to their purchase was heavily affected by the psychological impact of the pandemic on the occurrence of “panic buying” during lockdown [30].

Interestingly, cheap price and the large amount of spare time to be spent cooking at home could have contributed to the high increase in purchases of fresh vegetables and fruits, flour and eggs, confirming similar upward trends for basic ingredients found in the literature [20,24,31]. By contrast, we observed a decrease in purchases of ready-to-eat vegetables, as already found [8,21], suggesting increased attention was spent transforming raw food into dishes, therefore limiting the purchase of ready-made products. This trend could have improved the diet quality of our sample, since the daily consumption of fruits and vegetables has become the main tool for prevention of cardiovascular disease, from the public health viewpoint worldwide [32]. Moreover, a high consumption of fruit and vegetables, if kept over time, could be related to lower frailty risk [33] and inversely associated with the risk of cardiovascular disease [34] and mortality [35].

However, a strong decrease in fresh fish purchases occurred in our sample, since it is one of the most perishable foods, characterised by a short shelf life and usually sold in street markets, which were mostly limited or closed during lockdown. Taken together, these factors could have led to this decrease, as shown in Spain [20]. The Italian annual per capita consumption of fish was estimated by European Commission at about 30.9 kg in 2017 [36]. We expect a reduction trend by 2020 that could have health consequences, if maintained in the future. Indeed, evidence confirms the salutary effects of fish consumption on the prevention of coronary artery disease, stroke and dementia [32] while showing an inverse association with the risk of all-cause mortality [37].

During lockdown, most people increased the overall amount of food purchases, while an increase in food sales during lockdown was reported in April 2020 (+18% compared to the same period in 2019, +3% compared to the previous month) [21] and during the entire lockdown [23]. Panic buying and the increase in purchases occurred also during past epidemics such as severe acute respiratory syndrome (SARS) [38]. During lockdown, stockpiling and sudden increases in purchases of food and even toilet paper have caused several problems to the retail sector all around the world, increasing concerns about shortages of non-perishable food products, contributing to the indirect, socioeconomic strong impact of coronavirus on sane people [30]. Indeed, the occurrence of impulse buying was related to an increase in food purchase in our sample, as well as having worked during lockdown or having children. Workers usually ate food cooked out of home in their workplace, but during lockdown bars and restaurants were closed, so they had to face new habits, increasing the amount of food purchases accounting for the introduction of their work meals. On the counterpart, similar mechanisms occurred in families with children, resulting in an increase in food consumed (and previously purchased) at home instead of school canteens, which were closed during the lockdown period. Finally, we observed a relation between a perceived change in diet quality and increased food purchase, resulting in an increased consumption of healthier or unhealthier foods. A study found that, during lockdown, both healthy and unhealthy foods recorded an increase in buying: unhealthy foods were purchased more often to cope with stress and improve the mood, whereas healthy foods were purchased extensively considering the aim of keeping healthy and in shape despite the lockdown-related restrictions, resulting in both cases in a change of perception of diet quality [20].

Most of the sample reduced household food waste production, confirming recent findings [14,16] about decrease in food waste production and increased use of the leftover food during lockdown. Similar behaviours in different samples toward food waste production indicate that their drivers are likely to be similar in many cultures [39], thereby the suitability of adopting means to reduce food waste from one country to another can be explored, as it is possible to learn from the experience of other countries. The rising leftover-use routines have shown to be strong contributors to food waste reduction [39] closely followed by shopping routines. During lockdown, shopping frequency in Italy strongly decreased [40], confirming our findings, and potentially affecting household food waste production. Indeed, a negative impact of frequency of food shopping on household food waste quantities was found [41], even in an Italian sample [42]. We found a relation between not working during lockdown and reduction in household food waste production that could be explained by the lack of out-of-home meals (that before lockdown were typically some form of gatherings in restaurants, pubs or cafes) by students and an increased attention towards the economic impact of waste by general population having lost their job during this period. A relation between out-of-home meals frequency and food waste production was found in the literature [43,44]. Interestingly, the occurrence of impulse buying was related to a non-reduction in household food waste production, confirming that impulsive purchases and buying foods that are not intended to be bought can affect food waste behaviours [45,46]. The relation between being on a diet and food waste reduction confirmed the negative impact of unplanned meals shopping on household food waste production [45,46]. Indeed, people on a diet follow a planned meals routine, resulting in a precise and well-organised shopping list and behaviour.

Shopping experience has deeply changed during lockdown. Supermarkets set a maximum number of inside customers, causing big queues up to 2 h [47]. Supermarkets reduced their opening hours and working days, closing “non-essential goods” sectors [48]. Therefore, customers might have felt less time available and pressure to shop quickly [6]. These factors are thought to have a role in enhancing impulse buying [49]. Interestingly, the occurrence of impulse buying during lockdown in our sample halved its prevalence, compared to the period before. The Italian government advice to reduce shopping frequency and to buy only necessary goods might have encouraged the extensive use of shopping lists among the population. Moreover, lockdown-related job insecurity may have played a role in restricting unnecessary purchases. People with worsened diet quality, low psychological well-being or occurrence of emotional overeating could have bought and consumed more indulgence and junk food as a coping strategy for the stressful situation, feeling then guilty. The purchase and consumption of these foods (rich in fats, sugars and calories) could explain the relation of these conditions with the occurrence of impulse buying and sense of guilt after purchase.

## 5. Strengths and Limitations

To date, this is the first study investigating both food choice and factors associated with increased food purchase, occurrence of impulse buying and reduction in household food waste production during lockdown among the Italian general population. Moreover, the investigation was performed a few days after the end of lockdown, in order to highlight well-established effects of the whole confinement period on our sample, instead of partial investigations on different lockdown phases, potentially underestimating different behaviours that could have come out in the last weeks. Our sample was large and composed of people from every region of the country, leading the extensive data to take into account the national perspective. Finally, validated tests were adopted to assess mental well-being and the occurrence of emotional overeating, resulting in a valuable occasion of investigation of mental health and nutrition issues during the COVID-19 pandemic in Italy. However, our study has some limitations. The online spread of the survey led to an opportunistic sampling. Moreover, females accounted for 70% of our sample. Nevertheless, similar gender prevalence was observed in different studies conducted online during the lockdown period [11,14]. Food purchases and perceived change of habits were assessed in a qualitative fashion, without being given the opportunity to further explore their connections. In addition, the self-reporting of items could have represented itself a limitation in terms of quality of data (e.g., lack of memory, over/under-reporting). Finally, due to the cross-sectional design of the study, it was not possible to infer causal relationships between variables.

## 6. Implications and Conclusions

Overall, the effects of lockdown on population appeared to be both positive and negative, depending on the context. Food purchase, consumption and household food waste production in our sample were affected mostly in a desirable way, as evidenced by results. More efforts for public health interventions are needed to keep these new habits in the future, leading to positive behaviours toward achieving a sustainable and healthy lifestyle. Conversely, the lockdown appeared to affect heavily on mental health among a critically high portion of our sample, resulting in low psychological well-being, higher risk of depression and occurrence of emotional overeating as a possible coping strategy. Since the adoption of new lockdowns in the future cannot be excluded, policymakers should take into consideration this public health perspective, since for these people, a new containment measure could cause further negative effects on their physical and mental health. Moreover, our results can suggest strategies to the food retail sector about food categories that should be primarily provided in the case of new confinements, such as starchy foods, eggs, fresh fruits and vegetables, dairy products, considering that many issues occurred during the first lockdown regarding stockpiling and scarce food supplies in shops. Finally, the pandemic encouraged the adoption of online grocery purchase to the Italian population, offering a modern and low-risk shopping method. These services should be strengthened, especially in the southern regions of Italy, in order to make providers more resilient and prepared to satisfy an increased demand for service in the critical period to come, characterised by social distancing and home working.

## Figures and Tables

**Table 1:** Participant characteristics stratified by geographical area: Descriptive and Chi-square analysis.

Median [IQR] or n (%)						
Variables		All	North	Centre	South and Isles	*p*-Value
		(*n* = 1865)	(*n* = 927)	(*n* = 593)	(*n* = 345)	
Geographical area	North	927 (49.7)				
	Centre	593 (31.8)				
	South and Isles	345 (18.5)				
Age		29 [16.0]	29 [17.0]	29 [15.0]	27 [16.0]	**<0.001**
Gender	Female	1304 (69.9)	679 (73.2)	394 (66.4)	231 (67.0)	**0.021**
	Male	558 (29.2)	246 (26.5)	199 (33.6)	113 (32.8)	
	Non-Binary	3 (0.2)	2 (0.2)	0 (0.0)	1 (0.3)	
Relationship status *Missing = 5*	Into stable relationship or married	1194 (64.2)	600 (64.9)	387 (65.5)	207 (60.0)	0.193
	Single/divorced/widow	666 (35.8)	324 (35.1)	204 (34.5)	138 (40.0)	
Education level	Primary/Middle Sch.	87 (4.6)	45 (4.9)	16 (2.7)	26 (7.5)	**0.008**
	High School	803 (43.1)	397 (42.8)	246 (41.5)	160 (46.4)	
	University degree	785 (42.1)	387 (41.7)	265 (44.7)	133 (38.6)	
	Post-graduate ed.	190 (10.2)	98 (10.6)	66 (11.1)	26 (7.5)	
Living condition	Not alone	1647 (88.3)	794 (85.7)	538 (90.7)	315 (91.3)	**0.002**
	Alone	218 (11.7)	133 (14.3)	55 (9.3)	30 (8.7)	
Offspring	No	1441 (77.3)	709 (76.5)	468 (78.9)	264 (76.5)	0.508
	Yes	424 (22.7)	218 (23.5)	125 (21.1)	81 (23.5)	
Housing *Missing = 1*	Room	117 (6.3)	45 (4.9)	55 (9.3)	17 (4.9)	**0.005**
	Flat	1133 (60.8)	572 (61.7)	359 (60.5)	202 (58.6)	
	Independent house	615 (32.9)	308 (33.2)	179 (30.2)	123 (35.7)	
Yard/garden	Yes	618 (33.1)	313 (33.8)	181 (30.5)	124 (35.9)	0.200
	No	1247 (66.9)	614 (66.2)	412 (69.5)	221 (64.1)	
Working during lockdown	Working	773 (41.4)	431 (46.5)	237 (40.0)	104 (30.1)	**<0.001**
	Not working	1093 (58.6)	496 (53.5)	356 (60.0)	241 (69.9)	
Healthcare worker	Yes	342 (18.3)	188 (20.3)	105 (17.7)	49 (14.2)	**0.040**
	No	1523 (81.7)	739 (79.7)	488 (82.3)	296 (85.8)	
Smoke habit	Yes	409 (21.9)	182 (19.6)	150 (25.3)	77 (22.3)	**0.033**
	No	1456 (78.1)	745 (80.4)	443 (74.7)	268 (77.7)	
WHO-5 Well-being	≤50	1074 (57.6)	512 (55.2)	348 (58.7)	214 (62.0)	0.075
	>50	791 (42.4)	415 (44.8)	245 (41.3)	131 (38.0)	
EOQ-5	At risk	920 (49.3)	435 (46.9)	299 (50.4)	186 (53.9)	0.070
	Not at risk	945 (50.7)	492 (53.1)	294 (49.6)	159 (46.1)	
BMI *Missing = 10*	*Underweight*	118 (6.4)	65 (7.0)	39 (6.6)	14 (4.1)	0.203
	*Normal*	1273 (68.6)	638 (69.0)	391 (66.5)	244 (71.3)	
	*Overweight*	366 (19.7)	173 (18.7)	131 (22.3)	62 (19.2)	
	*Obese*	98 (5.3)	49 (5.3)	27 (4.6)	22 (6.4)	
Sport during lockdown	*Yes*	1220 (65.4)	612 (66.0)	391 (65.9)	217 (62.9)	0.553
	*No*	645 (34.6)	315 (34.0)	202 (34.1)	128 (37.1)	
Being on a diet during lockdown	*Yes*	444 (23.8)	218 (23.5)	143 (24.1)	83 (24.1)	0.958
	*No*	1421 (76.2)	709 (76.5)	450 (75.9)	262 (75.9)	

Abbreviations: IQR, interquartile range; N, number; Who-5, 5-item World Health Organization Well-Being Index (WHO-5) questionnaire; EOQ-5, Emotional Overeating Questionnaire-5 (EOQ-5); BMI, body mass index. In order to enhance readability, *p*-values < 0.05 are shown bolded.

**Table 2:** Food purchase and consumption habits stratified by geographical area: Descriptive and Chi-square analysis.

Median (IQR) or n (%)						
Variables		All (*n* = 1865)	North (*n* = 927)	Centre (*n* = 593)	South and Isles (*n* = 345)	*p*-Value
Food purchase	Decreased	134 (7.2)	54 (5.8)	50 (8.4)	30 (8.7)	0.090
	Unvaried	735 (39.4)	359 (38.7)	229 (38.6)	147 (42.6)	
	Increased	996 (53.4)	514 (55.4)	314 (53.0)	168 (48.7)	
Food consumption	Decreased	237 (12.7)	132 (14.2)	65 (11.0)	40 (11.6)	0.130
	Unvaried	818 (43.9)	402 (43.4)	270 (45.5)	138 (40.0)	
	Increased	810 (43.4)	393 (42.4)	258 (43.5)	167 (48.4)	
Perceived nutrition quality	Less healthy	502 (26.9)	230 (24.8)	161 (27.2)	111 (32.2)	0.079
	Unvaried	495 (26.6)	441 (47.6)	270 (45.5)	157 (45.5)	
	Healthier	868 (46.5)	256 (27.6)	162 (27.3)	77 (22.3)	
Household food waste production	Decreased	1002 (53.7)	500 (53.9)	315 (53.1)	187 (54.2)	0.933
	Unvaried	800 (42.9)	399 (43.0)	256 (43.2)	145 (42.0)	
	Increased	63 (3.4)	28 (3.0)	22 (3.7)	13 (3.8)	
Time spent cooking	Decreased	184 (9.9)	82 (8.8)	53 (8.9)	49 (14.2)	**<0.001**
	Unvaried	654 (35.0)	305 (32.9)	206 (34.7)	143 (41.4)	
	Increased	1027 (55.1)	540 (58.3)	334 (56.3)	153 (44.3)	
Grocery shopping	Personally	1513 (81.1)	772 (83.3)	483 (81.5)	258 (74.8)	**0.003**
	Someone for me	352 (18.9)	155 (16.7)	110 (18.5)	87 (25.2)	
N° trips for shopping	1/week or less	1285 (68.9)	669 (72.2)	400 (67.5)	216 (62.6)	**0.003**
	>1 per week	580 (31.1)	258 (27.8)	193 (32.5)	129 (37.4)	
	mean (SD)	1.51 (1.3)	1.42 (1.2)	1.55 (1.3)	1.65 (1.4)	**0.001**
Impulse buying during lockdown	No	1476 (79.1)	740 (79.8)	464 (78.2)	272 (78.8)	0.752
	Yes	389 (20.9)	187 (20.2)	129 (21.8)	73 (21.2)	
Impulse buying before lockdown	No	1073 (57.5)	554 (59.8)	326 (55.0)	193 (55.9)	0.147
	Yes	792 (42.5)	373 (40.2)	267 (45.0)	152 (44.1)	
Delivery food	No	1556 (83.4)	753 (81.2)	499 (84.1)	304 (88.1)	**0.011**
	Yes	309 (16.6)	174 (18.8)	94 (15.9)	41 (11.9)	
Food shops *	Supermarket	1635 (87.7)	806 (86.9)	543 (91.6)	297 (86.1)	0.171
	Small shops	711 (38.1)	452 (48.8)	231 (39.0)	128 (37.1)	0.846
	Discount market	306 (16.4)	133 (14.3)	110 (18.5)	63 (18.3)	0.057
	Online shops	277 (14.9)	172 (18.6)	74 (12.5)	31 (9.0)	**<0.001**
	Market	162 (8.7)	100 (10.8)	55 (9.3)	7 (2.0)	**<0.001**

* For this question, multiple answers were allowed. In order to enhance readability, *p*-values < 0.05 are shown bolded.

**Table 3:** Multivariable analysis: determinants of self-perceived changes of food purchase, household food waste production and occurrence of impulse buying.

Variables		Increased Food Purchase		Impulse Buying		Decreased Household Food Waste Production	
		*p*-Value	OR (IC 95%)	*p*-Value	OR (IC 95%)	*p*-Value	OR (IC 95%)
Age		**0.002**	0.98 (0.97–0.99)	0.115	0.99 (0.98–1)	**0.006**	**1.01 (1–1.02)**
Gender	Female	Ref		Ref		Ref	
	Male	0.055	0.81 (0.65–1)	**0.219**	0.83 (0.63–1.11)	**0.002**	**0.59 (0.47–0.74)**
Education	High	Ref		Ref		Ref	
	Med-Low	0.644	1.05 (0.85–1.31)	0.302	0.88 (0.68–1.13)	0.602	0.94 (0.76–1.17)
Sentimental status	Not single	Ref				Ref	
	Single	**0.028**	0.78 (0.62–0.97)			0.074	0.82 (0.66–1.02)
Offspring	No	Ref					
	Yes	**0.001**	1.76 (1.25–2.47)				
Cohabitation	Yes			Ref			
	No			**<0.002**	1.89 (1.32–2.71)		
Working during lockdown	Yes	Ref				Ref	
	No	**0.003**	0.71 (0.57–0.89)			**0.024**	1.3 (1.03–1.62)
Smoking habit	No	Ref				Ref	
	Yes	0.069	1.25 (0.98–1.58)			**<0.001**	1.8 (1.42–2.29)
BMI score	Normal			Ref		Ref	
	Underw.			0.318	0.76 (0.44–1.3)	**0.009**	0.71 (0.48–1.06)
	Overw.			**0.024**	1.44 (1.05–1.96)	**0.017**	1.37 (1.06–1.77)
	Obese			0.367	1.27 (0.75–2.15)	**0.034**	1.63 (1.04–2.57)
Time spent cooking	Unvaried	Ref		Ref		Ref	
	Decreased	0.192	0.79 (0.55–1.13)	**0.039**	1.58 (1.02–2.45)	**0.056**	1.41 (0.99–2)
	Increased	**<0.001**	2.12 (1.71–2.61)	**0.034**	1.36 (1.02–1.8)	**<0.001**	1.52 (1.23–1.88)
Perceived nutrition quality	Unvaried	Ref		Ref		Ref	
	Less Healthy	**<0.001**	1.66 (1.3–2.12)	**<0.001**	2.22 (1.68–2.93)	**0.001**	1.37 (1.08–1.75)
	Healthier	**0.033**	1.29 (1.02–1.63)	**0.196**	0.8 (0.57–1.12)	**<0.001**	2.27 (1.77–2.9)
EOQ Score	Not at risk			Ref			
	At risk			**<0.001**	1.68 (1.29–2.19)		
Dietary regimen during lockdown	No					Ref	
	Yes					**0.043**	0.79 (0.62–0.99)
WHO-5 Score	> 50			Ref		Ref	
	≤ 50			**<0.001**	1.73 (1.32–2.27)	**0.002**	0.72 (0.59–0.89)
Household food waste production	Unvar. or increased			Ref			
	Decreased			**0.012**	0.73 (0.57–0.93)		
Impulse buying	No	Ref				Ref	
	Yes	**<0.001**	2.48 (1.91–3.22)			**0.013**	0.73 (0.57–0.94)
Food purchase	Unvaried			Ref		Ref	
	Decreased			**0.223**	1.4 (0.82–2.4)	**0.011**	1.69 (1.13–2.53)
	Increased			**<0.001**	2.72 (2.05–3.62)	**0.221**	1.14 (0.92–1.41)

Each column refers to a binary logistic regression model. Empty boxes refer to variables excluded using stepwise backward selection. Abbreviations: IQR, interquartile range; N, number; Who-5, 5-item World Health Organization Well-Being Index (WHO-5) questionnaire; EOQ-5, Emotional Overeating Questionnaire-5 (EOQ-5); BMI, body mass in-dex. In order to enhance readability, *p*-values < 0.05 are shown bolded.

## Data Availability

The datasets generated and/or analysed during the current study are not publicly available due data are not public but are available from the corresponding author on reasonable request.

## Supplementary Materials

The following are available online at https://www.mdpi.com/2304-8158/10/2/306/s1, Document S1: Full regression outcomes; Document S3: Brief version of the questionnaire used for the survey; Image S2: Purchases per individual category.

## References

[1] Gazzetta Ufficiale DECRETO DEL PRESIDENTE DEL CONSIGLIO DEI MINISTRI 23 febbraio 2020. https://www.gazzettaufficiale.it/eli/id/2020/02/23/20A01228/sg.

[2] Gazzetta Ufficiale DECRETO DEL PRESIDENTE DEL CONSIGLIO DEI MINISTRI 1 marzo 2020. https://www.gazzettaufficiale.it/eli/id/2020/03/01/20A01381/sg.

[3] Gazzetta Ufficiale DECRETO DEL PRESIDENTE DEL CONSIGLIO DEI MINISTRI 9 marzo 2020. https://www.gazzettaufficiale.it/eli/id/2020/03/09/20A01558/sg.

[4] Vicentini C., Bordino V., Gardois P., Zotti C.M. (2020). Early assessment of the impact of mitigation measures on the COVID-19 outbreak in Italy. Public Health.

[5] Gazzetta Ufficiale DECRETO DEL PRESIDENTE DEL CONSIGLIO DEI MINISTRI 10 aprile 2020. https://www.gazzettaufficiale.it/eli/id/2020/04/11/20A02179/sg.

[6] Martin-Neuninger R., Ruby M.B. (2020). What Does Food Retail Research Tell Us About the Implications of Coronavirus (COVID-19) for Grocery Purchasing Habits?. Front. Psychol..

[7] IRI COVID-19: LA SPESA PER LARGO CONSUMO NELLA FASE ACUTA DELLA CRISI. https://www.iriworldwide.com/it-it/insights/publications/covid-19-la-spesa-per-largo-consumo-nella-fase-acu.

[8] ISMEA Emergenza COVID–19: Rapporto sulla domanda e l’offerta dei prodotti alimentari nelle prime settimane di diffusione del virus. http://www.ismea.it/flex/cm/pages/ServeBLOB.php/L/IT/IDPagina/11018.

[9] Altroconsumo Fare La Spesa Online Ai Tempi Del Coronavirus: Non Pochi Problemi. https://www.altroconsumo.it/alimentazione/fare-la-spesa/news/coronavirus-spesa-online.

[10] Haddad C., Kheir M.B., Zakhour M., Haddad R., Al Hachach M., Sacre H., Salameh P. (2020). Association between eating behavior and quarantine/confinement stressors during the Coronavirus disease 2019 outbreak. J. Eat. Disord..

[11] Cancello R., Soranna D., Zambra G., Zambon A., Invitti C. (2020). Determinants of the lifestyle changes during covid-19 pandemic in the residents of northern italy. Int. J. Environ. Res. Public Health.

[12] Coldiretti Istat, 2 kg di Peso Per il Lockdown a Tavola. https://www.coldiretti.it/salute-e-sicurezza-alimentare/istat-2-kg-di-peso-per-il-lockdown-a-tavola.

[13] Di Renzo L., Gualtieri P., Cinelli G., Bigioni G., Soldati L., Attinà A., Bianco F.F., Caparello G., Camodeca V., Carrano E. (2020). Psychological aspects and eating habits during covid-19 home confinement: Results of ehlc-covid-19 italian online survey. Nutrients.

[14] Di Renzo L., Gualtieri P., Pivari F., Soldati L., Attinà A., Cinelli G., Cinelli G., Leggeri C., Caparello G., Barrea L. (2020). Eating habits and lifestyle changes during COVID-19 lockdown: An Italian survey. J. Transl. Med..

[15] Sidor A., Rzymski P. (2020). Dietary choices and habits during COVID-19 lockdown: Experience from Poland. Nutrients.

[16] Jribi S., Ben Ismail H., Doggui D., Debbabi H. (2020). COVID-19 virus outbreak lockdown: What impacts on household food wastage?. Environ. Dev. Sustain..

[17] Ministero della Salute Come fare la spesa ai tempi del Covid-19 e Rispettare le Corrette Prassi di Igiene Alimentare. http://www.salute.gov.it/portale/news/p3_2_1_1_1.jsp?lingua=italiano&menu=notizie&p=null&id=4299.

[18] Topp C.W., Østergaard S.D., Søndergaard S., Bech P. (2015). The WHO-5 well-being index: A systematic review of the literature. Psychother. Psychosom..

[19] Casu G., Gremigni P., Masheb R.M. (2019). Emotional overeating questionnaire: A validation study in Italian adults with obesity, overweight or normal weight. Eat. Weight Disord..

[20] Laguna L., Fiszman S., Puerta P., Chaya C., Tárrega A. (2020). The impact of COVID-19 lockdown on food priorities. Results from a preliminary study using social media and an online survey with Spanish consumers. Food Qual. Prefer..

[21] ISMEA Emergenza COVID–19: 2° Rapporto Sulla Domanda e L’offerta dei Prodotti Alimentari Nell’emergenza Covid-19. http://www.ismea.it/flex/cm/pages/ServeBLOB.php/L/IT/IDPagina/11017.

[22] Centro di Ricerche su Retailing e Trade Marketing dell’Università Cattolica Come Cambia il Consumatore Dopo il Covid. https://www.cattolicanews.it/come-cambia-il-consumatore-dopo-il-covid.

[23] Bertoletti C. Nielsen Le Vendite in Gdo Durante il Lockdown: +4,2% Con Liberi Servizi al Traino. Gdoweek. https://www.gdoweek.it/le-vendite-in-gdo-durante-il-lockdown-42-con-liberi-servizi-al-traino/.

[24] Zhao A., Li Z., Ke Y., Huo S., Ma Y., Zhang Y., Zhang J., Ren Z. (2020). Dietary diversity among chinese residents during the COVID-19 outbreak and its associated factors. Nutrients.

[25] Mills S., Brown H., Wrieden W., White M., Adams J. (2017). Frequency of eating home cooked meals and potential benefits for diet and health: Cross-sectional analysis of a population-based cohort study. Int. J. Behav. Nutr. Phys. Act..

[26] Bracale R., Vaccaro C.M. (2020). Changes in food choice following restrictive measures due to Covid-19. Nutr. Metab. Cardiovasc. Dis..

[27] Macht M., Mueller J. (2007). Immediate effects of chocolate on experimentally induced mood states. Appetite.

[28] Gualano M.R., Lo Moro G., Voglino G., Bert F., Siliquini R. (2020). Effects of COVID-19 lockdown on mental health and sleep disturbances in Italy. Int. J. Environ. Res. Public Health.

[29] Ready Suggested Emergency Food Supplies. https://www.ready.gov/food.

[30] Nicola M., Alsafi Z., Sohrabi C., Kerwan A., Al-Jabir A., Iosifidis C., Agha M., Agha R. (2020). The Socio-Economic Implications of the Coronavirus Pandemic (COVID-19): A Review. Int. J. Surg..

[31] Batlle-Bayer L., Aldaco R., Bala A., Puig R., Laso J., Margallo M., Vázquez-Rowe I., Antó J.M., Fullana-i-Palmer P. (2020). Environmental and nutritional impacts of dietary changes in Spain during the COVID-19 lockdown. Sci. Total Environ..

[32] Román G.C., Jackson R.E., Gadhia R., Román A.N., Reis J. (2019). Mediterranean diet: The role of long-chain ω-3 fatty acids in fish; polyphenols in fruits, vegetables, cereals, coffee, tea, cacao and wine; probiotics and vitamins in prevention of stroke, age-related cognitive decline, and Alzheimer disease. Rev. Neurol. (Paris).

[33] Kojima G., Avgerinou C., Iliffe S., Jivraj S., Sekiguchi K., Walters K. (2018). Fruit and Vegetable Consumption and Frailty: A Systematic Review. J. Nutr. Heal. Aging.

[34] Zhan J., Liu Y.J., Cai L.B., Xu F.R., Xie T., He Q.Q. (2017). Fruit and vegetable consumption and risk of cardiovascular disease: A meta-analysis of prospective cohort studies. Crit. Rev. Food Sci. Nutr..

[35] Wang X., Ouyang Y., Liu J., Zhu M., Zhao G., Bao W., Hu F.B. (2014). Fruit and vegetable consumption and mortality from all causes, cardiovascular disease, and cancer: Systematic review and dose-response meta-analysis of prospective cohort studies. BMJ.

[36] European Commission Consumption of Fisheries and Aquaculture Products (2017). https://ec.europa.eu/fisheries/6-consumption_en.

[37] Wan Y., Zheng J., Wang F., Li D. (2017). Fish, long chain omega-3 polyunsaturated fatty acids consumption, and risk of all-cause mortality: A systematic review and dose-response meta-analysis from 23 independent prospective cohort studies. Asia Pac. J. Clin. Nutr..

[38] Ding H. (2009). Rhetorics of Alternative Media in an Emerging Epidemic: SARS, Censorship, and Extra-Institutional Risk Communication. Tech. Commun. Q..

[39] Stancu V., Haugaard P., Lähteenmäki L. (2016). Determinants of consumer food waste behaviour: Two routes to food waste. Appetite.

[40] ISMEA Emergenza COVID–19: 3° Rapporto Sulla Domanda e L’offerta Dei Prodotti Alimentari Nell’emergenza Covid-19. http://www.ismea.it/flex/cm/pages/ServeBLOB.php/L/IT/IDPagina/11116.

[41] Giordano C., Alboni F., Cicatiello C., Falasconi L. (2019). Do discounted food products end up in the bin? An investigation into the link between deal-prone shopping behaviour and quantities of household food waste. Int. J. Consum. Stud..

[42] Jörissen J., Priefer C., Bräutigam K.R. (2015). Food waste generation at household level: Results of a survey among employees of two European research centers in Italy and Germany. Sustainability.

[43] Ponis S.T., Papanikolaou P.A., Katimertzoglou P., Ntalla A.C., Xenos K.I. (2017). Household food waste in Greece: A questionnaire survey. J. Clean. Prod..

[44] Breda M., Hong-Bo L. (2017). ‘Waste not, want not’: Exploring green consumers’ attitudes towards wasting edible food and actions to tackle food waste. Br. Food J..

[45] Stefan V., van Herpen E., Tudoran A.A., Lähteenmäki L. (2013). Avoiding food waste by Romanian consumers: The importance of planning and shopping routines. Food Qual. Prefer..

[46] Bravi L., Francioni B., Murmura F., Savelli E. (2020). Factors affecting household food waste among young consumers and actions to prevent it. A comparison among UK, Spain and Italy. Resour. Conserv. Recycl..

[47] Il Fatto Quotidiano Coronavirus, a Milano Lunghe Code Davanti Ai Supermercati: In Fila Dalle 6.30 Del Mattino. Attese anche di 2 ore. https://www.ilfattoquotidiano.it/2020/03/21/coronavirus-a-milano-lunghe-code-davanti-ai-supermercati-in-fila-dalle-6-30-del-mattino-attese-anche-di-2-ore-le-immagini/5744340/.

[48] Nardi V. La Spesa ai Tempi del Coronavirus: Supermercati Chiusi la Domenica, Orari Ridotti e Vendita Solo di Beni di Prima Necessità. Il fatto alimentare. https://ilfattoalimentare.it/coronavirus-supermercati-orari.html.

[49] Hausman A. (2000). A multi-method investigation of consumer motivations in impulse buying behavior. J. Consum. Mark..

