# Supplementary material for: COVID-19 Lockdown and Self-Perceived Changes of Food Choice, Waste, Impulse Buying and Their Determinants in Italy: QuarantEat, a Cross-Sectional Study
Source: Foods. 2021 Feb 2;10(2):306. doi: 10.3390/foods10020306 (PMC7913081; doi:10.3390/foods10020306)
Supplement: Supplementary file 1 [file foods-10-00306-s001.zip › Supplementary Materials - Food Purchases.docx]

**Covid-19 lockdown and self-perceived changes of food choice, waste, impulse buying and their determinants in Italy: QuarantEat, a cross-sectional study**

**Alessandro Scacchi ^1^, Dario Catozzi ^1^, Edoardo Boietti ^1^, Fabrizio Bert ^1, 2^, Roberta Siliquini ^1, 2^**

^1^ Department of Public Health Sciences and Paediatrics, University of Torino, 10124 Torino, Italy; alessandro.scacchi@unito.it (A.S); dario.catozzi@unito.it (D.C); edoardo.boietti@unito.it (E.B); fabrizio.bert@unito.it (F.B.); roberta.siliquini@unito.it (R.S.)

^2^ Azienda Ospedaliero-Universitaria, City of Health and Science of Turin, 10126 Torino, Italy

* Correspondence: fabrizio.bert@unito.it

SUPPLEMENTARY MATERIAL

Purchases per Individual Food Category

*Orange*: decreased purchase

*Yellow*: stable purchase

*Green*: increased purchase

*Grey*: never purchased

*Orange*: decreased purchase; *yellow*: stable purchase; *green*: increased purchase; *grey*: never purchased

*Orange*: decreased purchase; *yellow*: stable purchase; *green*: increased purchase; *grey*: never purchased

*Orange*: decreased purchase; *yellow*: stable purchase; *green*: increased purchase; *grey*: never purchased
