# Supplementary material for: COVID-19 Lockdown and Self-Perceived Changes of Food Choice, Waste, Impulse Buying and Their Determinants in Italy: QuarantEat, a Cross-Sectional Study
Source: Foods. 2021 Feb 2;10(2):306. doi: 10.3390/foods10020306 (PMC7913081; doi:10.3390/foods10020306)
Supplement: Supplementary file 1 [file foods-10-00306-s001.zip › Supplementary Materials - Regressions.docx]

**Covid-19 lockdown and self-perceived changes of food choice, waste, impulse buying and their determinants in Italy: QuarantEat, a cross-sectional study**

**Alessandro Scacchi ^1^, Dario Catozzi ^1^, Edoardo Boietti ^1^, Fabrizio Bert ^1, 2^, Roberta Siliquini ^1, 2^**

^1^ Department of Public Health Sciences and Paediatrics, University of Torino, 10124 Torino, Italy; alessandro.scacchi@unito.it (A.S); dario.catozzi@unito.it (D.C); edoardo.boietti@unito.it (E.B); fabrizio.bert@unito.it (F.B.); roberta.siliquini@unito.it (R.S.)

^2^ Azienda Ospedaliero-Universitaria, City of Health and Science of Turin, 10126 Torino, Italy

* Correspondence: fabrizio.bert@unito.it

SUPPLEMENTARY MATERIAL

Full Regressions Outcomes

Each multivariable logistic regression model shows two phases:

- First phase: the most comprehensive model, with all variables included
- Second phase: shows the last phase of variables selection through a stepwise backwards model.

Age, gender and education were set as constant terms and therefore retained into the final model.

Missing values were excluded in a pairwise fashion.

Sign. = statistical significance expressed as *p-value* (threshold 0.05)

AdjOR = Adjusted Odds Ratio

AdjOR CI95% = 95% Confidence Interval of the Adjusted Odds Ratio, expressed inferior (Inf) and superior (Sup) limit

Ref = reference value

Increased food purchase predictors

|  |  | INCREASED FOOD PURCHASE | | | | | | | | |
| --- | --- | --- | --- | --- | --- | --- | --- | --- | --- | --- |
|  |  | Sign | AdjOR | AdjOR CI_95%_ | | Sign | AdjOR | AdjOR CI_95%_ | |  |
|  |  |  |  | Inf | Sup |  |  | Inf | Sup |  |
| **Age** |  | **0.017** | 0.986 | 0.975 | 0.997 | **<0.001** | 0.982 | 0.972 | 0.993 |  |
| **Gender** | Female | *Ref* | | | | *Ref* | | | | |
|  | Male | 0.307 | 0.888 | 0.708 | 1.115 | 0.055 | 0.810 | 0.653 | 1.004 |  |
| **Education** | High | *Ref* | | | | *Ref* | | | | |
|  | Medium-Low | 0.606 | 1.062 | 0.845 | 1.335 | 0.644 | 1.053 | 0.846 | 1.310 |  |
| **Geography** | North | *Ref* | | | |  |  |  |  |  |
|  | Center | 0.124 | 0.809 | 0.617 | 1.060 |  |  |  |  |  |
|  | South and Isles | 0.430 | 0.914 | 0.730 | 1.143 |  |  |  |  |  |
| **Sentimental status** | Not single | *Ref* | | | | *Ref* | | | | |
|  | Single | 0.062 | 0.804 | 0.639 | 1.011 | **0.028** | 0.779 | 0.624 | 0.973 |  |
| **Offspring** | No | *Ref* | | | | *Ref* | | | | |
|  | Yes | **0.005** | 1.663 | 1.168 | 2.366 | **0.001** | 1.755 | 1.249 | 2.467 |  |
| **Cohabitation** | Yes | *Ref* | | | |  |  |  |  |  |
|  | No | 0.120 | 0.768 | 0.551 | 1.071 |  |  |  |  |  |
| **Working during lockdown** | Yes | *Ref* | | | | *Ref* | | | | |
|  | No | **0.015** | 0.739 | 0.580 | 0.943 | **0.003** | 0.712 | 0.567 | 0.893 |  |
| **Healthcare worker** | No | *Ref* | | | |  |  |  |  |  |
|  | Yes | 0.302 | 1.167 | 0.870 | 1.566 |  |  |  |  |  |
| **Smoking habit** | No | *Ref* | | | | *Ref* | | | | |
|  | Yes | 0.085 | 1.237 | 0.971 | 1.576 | 0.069 | 1.246 | 0.983 | 1.579 |  |
| **BMI score** | Normal | *Ref* | | | |  |  |  |  |  |
|  | Underweight | 0.870 | 1.035 | 0.688 | 1.555 |  |  |  |  |  |
|  | Overweight | 0.080 | 0.789 | 0.605 | 1.029 |  |  |  |  |  |
|  | Obese | 0.737 | 0.924 | 0.582 | 1.467 |  |  |  |  |  |
| **Time spent cooking** | Unvaried | *Ref* | | | | *Ref* | | | | |
|  | Increased | 0.173 | 0.779 | 0.544 | 1.116 | 0.192 | 0.790 | 0.554 | 1.126 |  |
|  | Diminished | **<0.001** | 2.115 | 1.706 | 2.621 | **<0.001** | 2.117 | 1.713 | 2.615 |  |
| **Perceived nutrition quality** | Unvaried | *Ref* | | | | *Ref* | | | | |
|  | Less healthy | **<0.001** | 1.626 | 1.263 | 2.095 | **<0.001** | 1.662 | 1.302 | 2.122 |  |
|  | Healthier | **0.016** | 1.351 | 1.057 | 1.726 | **0.033** | 1.291 | 1.020 | 1.633 |  |
| **EOQ Score** | Not at risk | *Ref* | | | |  |  |  |  |  |
|  | At risk | 0.171 | 1.159 | 0.938 | 1.432 |  |  |  |  |  |
| **Sport during lockdown** | No | *Ref* | | | |  |  |  |  |  |
|  | Yes | 0.675 | 0.953 | 0.762 | 1.193 |  |  |  |  |  |
| **Dietary regimen during lockdown** | No | *Ref* | | | |  |  |  |  |  |
|  | Yes | 0.630 | 0.943 | 0.743 | 1.197 |  |  |  |  |  |
| **WHO-5 Score** | > 50 | *Ref* | | | |  |  |  |  |  |
|  | ≤ 50 | 0.335 | 1.111 | 0.897 | 1.375 |  |  |  |  |  |
| **Food waste** | Same/increased | *Ref* | | | |  |  |  |  |  |
|  | Diminished | 0.658 | 1.047 | 0.854 | 1.284 |  |  |  |  |  |
| **Impulse buying during lockdown** | No | *Ref* | | | | *Ref* | | | | |
|  | Yes | **<0.001** | 2.474 | 1.895 | 3.228 | **<0.001** | 2.482 | 1.913 | 3.220 |  |

Impulse buying during lockdown predictors

|  |  | OCCURRENCE OF IMPULSE BUYING | | | | | | | |
| --- | --- | --- | --- | --- | --- | --- | --- | --- | --- |
|  |  | Sign | AdjOR | AdjOR CI_95%_ | | Sign | AdjOR | AdjOR CI_95%_ | |
|  |  |  |  | Inf | Sup |  |  | Inf | Sup |
| **Age** |  | 0.180 | 0.990 | 0.975 | 1.005 | 0.115 | 0.992 | 0.981 | 1.002 |
| **Gender** | Female | *Ref* | | | | *Ref* | | | |
|  | Male | 0.196 | 0.824 | 0.615 | 1.105 | 0.220 | 0.835 | 0.625 | 1.114 |
| **Education** | High | *Ref* | | | | *Ref* | | | |
|  | Medium-Low | 0.877 | 0.978 | 0.733 | 1.304 | 0.302 | 0.877 | 0.683 | 1.126 |
| **Geography** | North | *Ref* | | | |  |  |  |  |
|  | Center | 0.838 | 1.036 | 0.740 | 1.450 |  |  |  |  |
|  | South and Isles | 0.395 | 1.128 | 0.854 | 1.489 |  |  |  |  |
| **Sentimental status** | Not single | *Ref* | | | |  |  |  |  |
|  | Single | 0.231 | 0.838 | 0.628 | 1.119 |  |  |  |  |
| **Offspring** | No | *Ref* | | | |  |  |  |  |
|  | Yes | 0.651 | 0.906 | 0.590 | 1.391 |  |  |  |  |
| **Cohabitation** | Yes | *Ref* | | | | *Ref* | | | |
|  | No | **<0.001** | 1.943 | 1.315 | 2.871 | **<0.001** | 1.887 | 1.315 | 2.706 |
| **Working during lockdown** | Yes | *Ref* | | | |  |  |  |  |
|  | No | 0.532 | 0.907 | 0.666 | 1.234 |  |  |  |  |
| **Healthcare worker** | No | *Ref* | | | |  |  |  |  |
|  | Yes | 0.506 | 1.127 | 0.793 | 1.601 |  |  |  |  |
| **Smoking habit** | No | *Ref* | | | |  |  |  |  |
|  | Yes | 0.819 | 1.035 | 0.774 | 1.383 |  |  |  |  |
| **BMI score** | Normal | *Ref* | | | | *Ref* | | | |
|  | Underweight | 0.295 | 0.748 | 0.434 | 1.288 | 0.318 | 0.759 | 0.442 | 1.304 |
|  | Overweight | **0.020** | 1.457 | 1.061 | 2.000 | **0.024** | 1.435 | 1.050 | 1.962 |
|  | Obese | 0.418 | 1.246 | 0.731 | 2.123 | 0.367 | 1.273 | 0.753 | 2.152 |
| **Time spent cooking** | Unvaried | *Ref* | | | | *Ref* | | | |
|  | Increased | **0.041** | 1.580 | 1.019 | 2.452 | **0.039** | 1.583 | 1.023 | 2.451 |
|  | Diminished | 0.054 | 1.324 | 0.995 | 1.760 | **0.034** | 1.355 | 1.023 | 1.796 |
| **Perceived nutrition quality** | Unvaried | *Ref* | | | | *Ref* | | | |
|  | Less healthy | **<0.001** | 2.170 | 1.637 | 2.875 | **<0.001** | 2.221 | 1.681 | 2.934 |
|  | Healthier | 0.274 | 0.824 | 0.583 | 1.165 | 0.197 | 0.800 | 0.570 | 1.123 |
| **EOQ Score** | Not at risk | *Ref* | | | | *Ref* | | | |
|  | At risk | **<0.001** | 1.705 | 1.308 | 2.222 | **<0.001** | 1.680 | 1.291 | 2.186 |
| **Sport during lockdown** | No | *Ref* | | | |  |  |  |  |
|  | Yes | 0.621 | 0.934 | 0.712 | 1.225 |  |  |  |  |
| **Dietary regimen during lockdown** | No | *Ref* | | | |  |  |  |  |
|  | Yes | 0.389 | 0.871 | 0.637 | 1.192 |  |  |  |  |
| **WHO-5 Score** | > 50 | *Ref* | | | | *Ref* | | | |
|  | ≤ 50 | **<0.001** | 1.712 | 1.296 | 2.261 | **<0.001** | 1.730 | 1.316 | 2.275 |
| **Food Waste** | Same/increased | *Ref* | | | | *Ref* | | | |
|  | Diminished | **0.012** | 0.723 | 0.562 | 0.931 | **0.012** | 0.726 | 0.566 | 0.932 |
| **Food quantity purchase** | Unvaried | *Ref* | | | | *Ref* | | | |
|  | Decreased | 0.220 | 1.403 | 0.816 | 2.410 | 0.223 | 1.399 | 0.815 | 2.399 |
|  | Increased | **<0.001** | 2.689 | 2.019 | 3.579 | **<0.001** | 2.724 | 2.049 | 3.621 |

Diminished Food Waste predictors

|  |  | DIMINISHED FOOD WASTE | | | | | | | |
| --- | --- | --- | --- | --- | --- | --- | --- | --- | --- |
|  |  | Sign | AdjOR | AdjOR CI_95%_ | | Sign | AdjOR | AdjOR CI_95%_ | |
|  |  |  |  | Inf | Sup |  |  | Inf | Sup |
| **Age** |  | 0.095 | 1.010 | 0.998 | 1.021 | **0.006** | 1.012 | 1.003 | 1.020 |
| **Gender** | Female | *Ref* | | | | *Ref* | | | |
|  | Male | **<0.001** | 0.600 | 0.480 | 0.751 | **<0.001** | 0.591 | 0.474 | 0.739 |
| **Education** | High | *Ref* | | | | *Ref* | | | |
|  | Medium-Low | 0.368 | 0.902 | 0.719 | 1.130 | 0.602 | 0.944 | 0.762 | 1.171 |
| **Geography** | North | *Ref* | | | |  |  |  |  |
|  | Center | 0.532 | 1.089 | 0.834 | 1.422 |  |  |  |  |
|  | South and Isles | 0.644 | 0.949 | 0.761 | 1.184 |  |  |  |  |
| **Sentimental status** | Not single | *Ref* | | | | *Ref* | | | |
|  | Single | 0.106 | 0.829 | 0.661 | 1.041 | 0.074 | 0.822 | 0.663 | 1.019 |
| **Offspring** | No | *Ref* | | | |  |  |  |  |
|  | Yes | 0.394 | 1.162 | 0.823 | 1.641 |  |  |  |  |
| **Cohabitation** | Yes | *Ref* | | | |  |  |  |  |
|  | No | 0.915 | 1.018 | 0.732 | 1.416 |  |  |  |  |
| **Working during lockdown** | Yes | *Ref* | | | | *Ref* | | | |
|  | No | 0.087 | 1.234 | 0.970 | 1.571 | **0.024** | 1.296 | 1.034 | 1.625 |
| **Healthcare worker** | No | *Ref* | | | |  |  |  |  |
|  | Yes | 0.253 | 1.182 | 0.887 | 1.576 |  |  |  |  |
| **Smoking habit** | No | *Ref* | | | | *Ref* | | | |
|  | Yes | **<0.001** | 1.818 | 1.430 | 2.311 | **<0.001** | 1.803 | 1.421 | 2.287 |
| **BMI score** | Normal | *Ref* | | | | *Ref* | | | |
|  | Underweight | 0.126 | 0.731 | 0.489 | 1.093 | 0.093 | 0.709 | 0.475 | 1.059 |
|  | Overweight | **0.023** | 1.353 | 1.042 | 1.758 | **0.017** | 1.368 | 1.057 | 1.772 |
|  | Obese | **0.047** | 1.596 | 1.006 | 2.530 | **0.035** | 1.631 | 1.035 | 2.569 |
| **Time spent cooking** | Unvaried | *Ref* | | | | *Ref* | | | |
|  | Increased | 0.054 | 1.414 | 0.995 | 2.011 | 0.056 | 1.406 | 0.991 | 1.996 |
|  | Diminished | **<0.001** | 1.533 | 1.236 | 1.901 | **<0.001** | 1.520 | 1.228 | 1.883 |
| **Perceived nutrition quality** | Unvaried | *Ref* | | | | *Ref* | | | |
|  | Less Healthy | **0.022** | 1.335 | 1.043 | 1.708 | **0.010** | 1.374 | 1.080 | 1.749 |
|  | Healthier | **<0.001** | 2.264 | 1.768 | 2.899 | **<0.001** | 2.268 | 1.773 | 2.900 |
| **EOQ Score** | Not at risk | *Ref* | | | |  |  |  |  |
|  | At risk | 0.162 | 0.861 | 0.699 | 1.062 |  |  |  |  |
| **Sport during lockdown** | No | *Ref* | | | |  |  |  |  |
|  | Yes | 0.478 | 1.083 | 0.868 | 1.352 |  |  |  |  |
| **Dietary regimen during lockdown** | No | *Ref* | | | | *Ref* | | | |
|  | Yes | **0.031** | 0.771 | 0.608 | 0.977 | **0.043** | 0.786 | 0.622 | 0.993 |
| **WHO-5 Score** | > 50 | *Ref* | | | | *Ref* | | | |
|  | ≤ 50 | **0.001** | 0.709 | 0.574 | 0.876 | **0.002** | 0.725 | 0.591 | 0.889 |
| **Impulse buying during lockdown** | No | *Ref* | | | | *Ref* | | | |
|  | Yes | **0.011** | 0.724 | 0.564 | 0.930 | **0.013** | 0.731 | 0.570 | 0.937 |
| **Food quantity purchase** | Unvaried | *Ref* | | | | *Ref* | | | |
|  | Increased | **0.013** | 1.673 | 1.115 | 2.512 | **0.011** | 1.686 | 1.125 | 2.527 |
|  | Diminished | 0.251 | 1.133 | 0.916 | 1.401 | 0.221 | 1.141 | 0.924 | 1.410 |
