# Supplementary material for: COVID-19 Lockdown and Self-Perceived Changes of Food Choice, Waste, Impulse Buying and Their Determinants in Italy: QuarantEat, a Cross-Sectional Study
Source: Foods. 2021 Feb 2;10(2):306. doi: 10.3390/foods10020306 (PMC7913081; doi:10.3390/foods10020306)
Supplement: Supplementary file 1 [file foods-10-00306-s001.zip › Supplementary Materials - Survey.docx]

**Covid-19 lockdown and self-perceived changes of food choice, waste, impulse buying and their determinants in Italy: QuarantEat, a cross-sectional study**

**Alessandro Scacchi ^1^, Dario Catozzi ^1^, Edoardo Boietti ^1^, Fabrizio Bert ^1, 2^, Roberta Siliquini ^1, 2^**

^1^ Department of Public Health Sciences and Paediatrics, University of Torino, 10124 Torino, Italy; alessandro.scacchi@unito.it (A.S); dario.catozzi@unito.it (D.C); edoardo.boietti@unito.it (E.B); fabrizio.bert@unito.it (F.B.); roberta.siliquini@unito.it (R.S.)

^2^ Azienda Ospedaliero-Universitaria, City of Health and Science of Turin, 10126 Torino, Italy

* Correspondence: fabrizio.bert@unito.it

SUPPLEMENTARY MATERIAL

Brief version of the questionnaire used for the survey

| **Question** | **Answers** |
| --- | --- |
| Gender | Male / Female / Non binary |
| Age |  |
| Height |  |
| Weight |  |
| City of residency |  |
| Nationality |  |
| Sentimental status | Single / In a relationship / Married / Divorced |
| Offspring presence | Yes, one / Yes, more than one / No |
| Cohabitation | Alone / Family / Partner / Roommates |
| Type of dwelling | Room / Flat / Independent house with garden |
| Education achieved | None / Primary school / Middle school / High school / Degree / PhD or post-degree education |
| Working status | Student / Unemployed / Retired / Homemaker / Employed / Freelance |
| Worked during lockdown | Yes / No |
| Healthcare worker | Yes / No |
| Type of work during lockdown | Workplace, full time / Workplace, part-time / Working from home |
| Received salary, layoff or subsidies | Yes / No |
| Smoking status | Yes / No |
| Tobacco consumption during lockdown | Increased / Unvaried / Decreased |
| During last 28 days:  I have felt cheerful and in good spirit / I have felt calm and relaxed / I have felt active and vigorous / I woke up feeling fresh and rested / my daily life has been filled with things that interest me | All of the time / Most of the time / More than half the time / Less than half the time / Some of the time / At no time |
| Time spent cooking / Snacking / Feeling of guilt after eating unhealthy or high-calories foods (fat or sweet foods) / Drinking alcohol with meals / Drinking alcohol out of meals / Drinking alcohol to relax / Drinking alcohol until drunk / Drinking coffee or caffeine-based products / Consumption of relaxing products (chamomile, melatonin) / Night-time or before bad snacks / Feeling of hunger during the day / Eating while staring at screens / Eating to relax or for boredom / Eating to reward or gratify myself | Frequency during lockdown compared to the period before:  Increased / Unvaried / Decreased |
| Frequency, over the prior 28 days, of overeating in response to anxiety, sadness, loneliness, tiredness, anger, and happiness | No days / 1–5 days / 6–12 days / 13–15 days / 16–22 days / 23–27 / Everyday |
| Physical activity during lockdown / Physical activity before lockdown / Purchase of sports equipment / Purchase of sports gear / Purchase of sports food | Yes / No |
| Training frequency (weekly) |  |
| Training modality | Contents on social media / Remote personal training / online gym workouts / maintaining usual training / independently |
| Made purchase during lockdown | Yes / No |
| Place of purchases | Market / Supermarket / Neighbourhood shop / Discount / Online shop / Food delivery |
| Shopping frequency (weekly) |  |
| Perceived food purchase quantity during lockdown, compared to the period before | Increased / Unvaried / Decreased |
| Occurrence of sense of guilt, unnecessary purchase or remorse after a purchase during lockdown | Yes / no |
| Occurrence of sense of guilt, unnecessary purchase or remorse after a purchase before lockdown | Yes / No |
| Dietary regimen or food exclusions during lockdown | Yes (which one) / No |
| Dietary regimen or food exclusions during lockdown | Yes (which one) / No |
| Perceived food consumption during lockdown | Increased / Unvaried / Decreased |
| Perceived dietary quality during lockdown | Healthier / Unvaried / Less healthy |
| Quantity of household food waste during lockdown (food thrown away or expired) | Increased / Unvaried / Decreased |
| Purchase of each food during lockdown:  Pasta / Flour - Yeast / Eggs / Bread / Fresh fruits / Fresh vegetables / Ready-to-eat vegetables / Frozen vegetables / Sauces and preserves / Canned legumes / Canned meat / Canned fish / Ready-made meals/ Whole-grain foods / km0 foods / Organic foods / Read meat / White meat / Fresh fish / Frozen fish / Cold cuts / Fresh cheese / Matured cheese / Frozen ready-made meals / Frozen bakery products / Fresh milk / UHT Milk / Yogurt / Biscuits / Sauces and dressings / Salted snacks / Chocolate / Creams and jams / Sweet snacks / Ice cream - cakes / Soft drinks / Fruit juice / Butter / Oil / Beer / Wine / Spirits / Supplements / Food delivery: Italian / Food delivery: Pizza / Food delivery: Burgers - rotisserie / Food delivery: ethnic food / Food delivery: soft drinks / Food delivery: alcoholic beverages / Food delivery: sweets and ice creams | Increased / Unvaried / Decreased |
